# Exploring and Selecting Features to Predict the Next Outcomes of MLB Games

**DOI:** 10.3390/e24020288

**Published:** 2022-02-17

**Authors:** Shu-Fen Li, Mei-Ling Huang, Yun-Zhi Li

**Affiliations:** Department of Industrial Engineering & Management, National Chin-Yi University of Technology, Taichung 41170, Taiwan; sfli@ncut.edu.tw (S.-F.L.); 4a915015@gm.student.ncut.edu.tw (Y.-Z.L.)

**Keywords:** Major League Baseball (MLB), model prediction, machine learning, deep learning

## Abstract

(1) Background and Objective: Major League Baseball (MLB) is one of the most popular international sport events worldwide. Many people are very interest in the related activities, and they are also curious about the outcome of the next game. There are many factors that affect the outcome of a baseball game, and it is very difficult to predict the outcome of the game precisely. At present, relevant research predicts the accuracy of the next game falls between 55% and 62%. (2) Methods: This research collected MLB game data from 2015 to 2019 and organized a total of 30 datasets for each team to predict the outcome of the next game. The prediction method used includes one-dimensional convolutional neural network (1DCNN) and three machine-learning methods, namely an artificial neural network (ANN), support vector machine (SVM), and logistic regression (LR). (3) Results: The prediction results show that, among the four prediction models, SVM obtains the highest prediction accuracies of 64.25% and 65.75% without feature selection and with feature selection, respectively; and the best AUCs are 0.6495 and 0.6501, respectively. (4) Conclusions: This study used feature selection and optimized parameter combination to increase the prediction performance to around 65%, which surpasses the prediction accuracies when compared to the state-of-the-art works in the literature.

## 1. Introduction

Sports events have been deeply connected into the lives of the general public. Among them, baseball is one of the most popular sports. Major League Baseball (MLB) is the world’s highest-level professional baseball game and has a long history in all North American professional sports leagues. A large amount of game data is open to the public, and many scholars have invested in the research field of predicting the outcome of the game, player performance, and player value. It is very fascinating and important to find out what the key variables are that affect the outcome of the game.

Barnes and Bjarnadóttir [1] collected player data from 1998 to 2014 and used linear regression (LR), random forest (RF), regression trees (RT), and gradient-boosted trees (GBT) to predict the wins above replacement (WAR) of players. WAR represents the indicator of how many victories a player can bring to the team, and it is then converted into the market value of the player. Sidle and Tran [2] collected pitcher competition data from 2013 to 2015 and used multi-class linear discriminant analysis, support vector machines (SVM), and decision trees (DTs) to predict next type of pitch; they developed a real-time and live-game predictor and finally achieved a real-time success rate of more than 60%.

Manoj et al. [3] collected American League (AL) game data that included four important factors, namely home/away, day/night, ranking, and division, using the Analytic Hierarchy Process (AHP), to predict the 2017 season champion. The result can show the winning probability for each team. For example, the AHP model predicts that the winning probability for Kansas City Royals (KCR) is 0.6106, and this team is the most likely team to become the 2017 season champion. Huang and Li [4] collected 2019 MLB game data, including hitting, pitcher, and home/away, to compare the prediction accuracies between using the data of the starting pitcher or the entire pitcher before and after the feature selection. The best prediction accuracy (94.18%) was obtained from an artificial neural network (ANN) after feature selection from the entire pitcher database.

Of the related studies predicting the MLB outcome of the next game, Jia et al. [5] collected team competition data from 2007 to 2012. They (1) applied multiple LR and RF to predict scores to further judge wins or losses and (2) used classification methods, including logistic regression, SVM, AdaBoost, and LogitBoost, to predict wins or losses. Results showed that SVM with the radial basis function (RBF) kernel obtains the best accuracy (59.60%). Elfrink [6] applied LR, RF, eXtreme Gradient Boosting (XGBoost), and Boosted LR to predict the MLB outcome for the next game by using the data from 1930 to 2016. To create a fair result, all statistics of data they used were based on data previous to the game date to make sure that all predictions used data that were generated before the actual game. The results showed that XGBoost achieved the highest accuracy, i.e., 55.52%.

Soto Valero [7] accumulated the game data of 10 regular seasons as a unit and used k-nearest-neighbor algorithm (K-NN), ANN, DT, and SVM to predict the outcome of the next game. Finally, SVM with sequential minimal optimization (SMO) algorithm obtained the best prediction accuracy (58.92%). Cui [8] sorted out the game data from 2000 to 2019, including data on the hitting and the starting pitcher, with a total of nine input variables to predict the outcome of the next game by using logistic regression, SVM, K-NN, DT, RF, and XGBoost. The best accuracy of 61.77% was from logistic regression.

A valuable research study differentiated player behaviors by gender, using data mining and polar coordinates analysis. Especially, this study mentioned the observational methodology and some important ethical principles to provide guidance to physicians and other participants in medical research involving human subjects [9].

Based on the abovementioned literature on predicting the outcome of the next MLB match, the accuracy falls between 55% and 62%. This study referred to the previous literature, using data accumulation methods, and applied ANN, SVM, logistic regression, and deep learning to predict the outcome of next MLB match.

## 2. Materials and Methods

### 2.1. Research Design

This study focused on predicting the outcome of the next MLB match for each team. Multiple dimensional data were collected from public platforms. The study was carried out by following the Belmont Report and was conducted in accordance with the Declaration of Helsinki (WMA 2000, Bošnjak 2001, Tyebkhan 2003). The observers did not interact with the subjects.

### 2.2. Participants

MLB game data of thirty teams for the 2015–2019 seasons were collected and analyzed. There are around 162 matches for each team in the mentioned 5 years. According to the Belmont Report, since all the data are open to the public, it is not required to obtain informed consent from the participants.

### 2.3. Data Preprocessing

MLB game data were collected by using the following steps. Step 1: We used Python PyBaseball2.2.0 (Python Software Foundation, Fredericksburg, Virginia, USA) [10] to download the game data for each team from Baseball-reference.com (assessed on 21 January 2021) to establish thirty datasets. Step 2: The data of each dataset were preprocessed. The game data of the *n*th game are the accumulation of the first *n* games, and the accumulation process is continued to the end of each season. After all accumulation processes were completed for the five seasons, all the datasets were normalized. Step 3: The recursive feature elimination (RFE) was applied for feature selection. Step 4: The dataset was split into training and testing sets with the ratio of 8:2.

**Figure 1:**
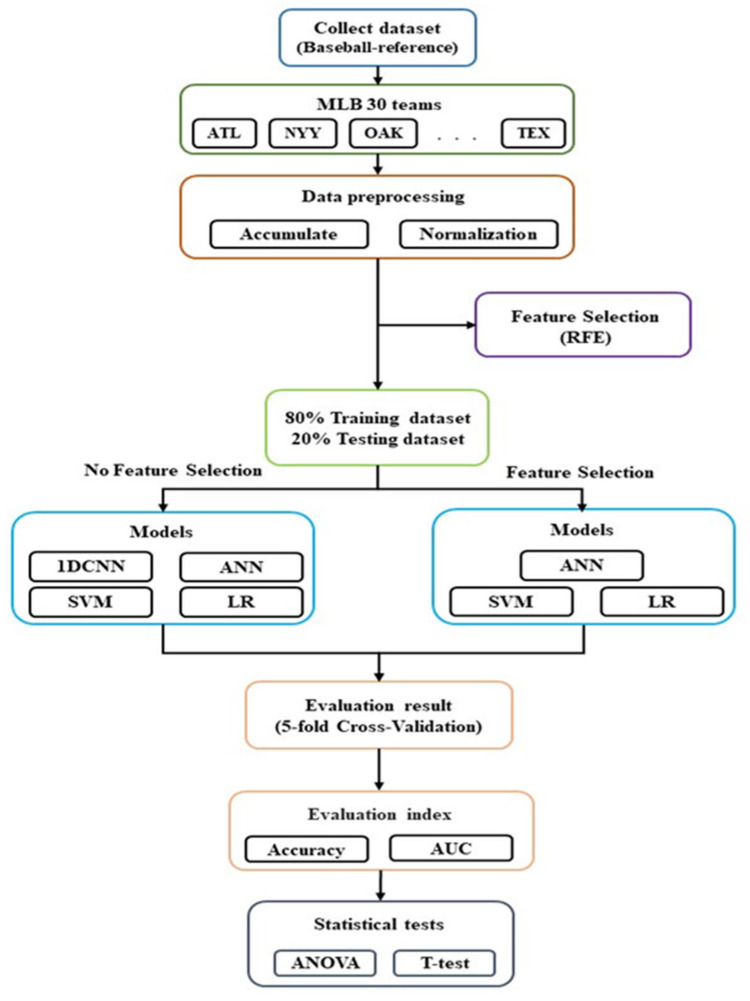
Flowchart of the study.

As illustrated in Figure 1, the original dataset before feature selection was treated as the input variables for ANN, SVM, LR, and one-dimensional convolutional neural network (1DCNN), while ANN, SVM, and LR were used for the dataset after feature selection. Five-fold cross-validation was used, and the area under curve (AUC) and accuracy were selected as the performance indicators to compare the performance for each model.

Many websites record the MLB game data, but the game data or variables recorded by each site are slightly different. For example, Retrosheet website (https://www.retrosheet.org (assessed on 21 January 2021)) and Lahman website (https://www.seanlahman.com (assessed on 21 January 2021)) record the original game data. Those original data can be processed into Sabermetrics. The Lahman website records the game data for the whole season, and there are no specific game data for each game; meanwhile, we can see detailed records of players, referees, managers, and weather for each game from Retrosheet and Baseball-reference websites. The Baseball-reference website (https://www.baseball-reference.com (assessed on 21 January 2021)) also provides Sabermetrics and other details, such as date, time (day/night), left or right hand for players, etc. It is user-friendly in regard to searching the game data for specific player or individual game data from the Baseball-reference website. Therefore, we selected the Baseball-reference website to collect game data in this study.

**Figure 2:**
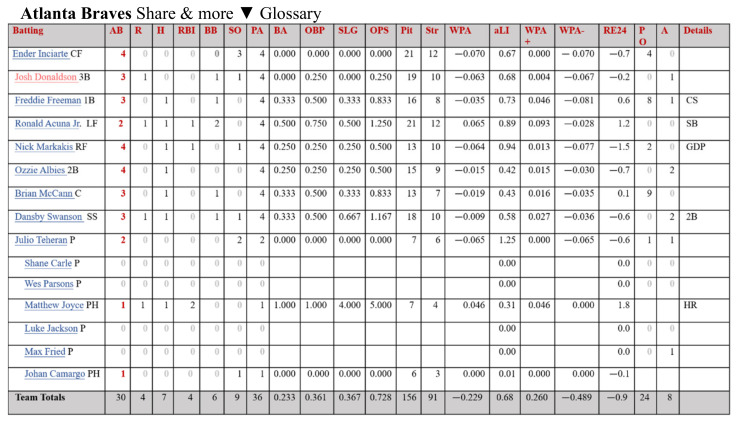
Hit-related variables from Baseball-reference.

**Figure 3:**
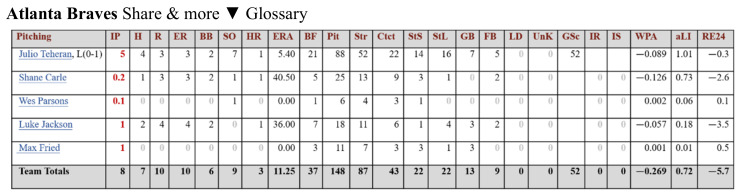
Pitcher-related variables from Baseball-reference.

The variables downloaded from Baseball-reference are divided into hits (Figure 2), pitcher performance (Figure 3), and scoring. Since the purpose of this research was to predict the outcome of the next game, more variables related to the outcome were selected, such as score (Run), scored (Runed), RBI (RBI), winning rate (*Win%*), etc. The winning rate (*Win%*) was calculated by this research as shown in Formula (1).

(1) $$The\;nth\;match\prime s\;Win\%=\frac{Number\;of\;wins\;in\;the\;first\;n\;matches}{n\;matches}$$

For each team, we collected 15 hit-related variables (B1~B15), 8 pitcher-related variables (P1~P8), and the *Win%* (*X1*)—24 variables in total, as displayed in Table 1. Accumulation was used for the next game. Take the Houston Astros team (HOU) as an example. In Table 2, the figures for game #3 in 2015 were the sum of games #1 to #3; the figures for game #162 in 2015 were the sum of games #1 to #162. *Y* represents the outcome of the next game. The figures were reset for each year. We repeated the process from year 2015 to 2019 to construct 30 datasets for 30 teams.

Before constructing the prediction models, variables in the 30 datasets were normalized by Min–Max normalization method to adjust each figure range between 0 and 1 as follows:

(2) $$X_{nom}=\frac{X-X_{min}}{X_{max}-X_{min}}\in \left[0,\;1\right]$$

$X_{max}$ is the maximum value, and $X_{min}$ is the minimum value for each variable.

### 2.4. Feature Selection

The main function of feature selection is to reduce redundant and unnecessary variables, thereby improving the prediction performance of the model. There are many methods for feature selection, and they are mainly divided into three categories: wrapper, filter, and embedding. This research used Recursive Feature Elimination (RFE), a feature-selection method belonging to the wrapper method. The main principle is to search for feature subsets from all the features in the training dataset, and then remove the less important features. Finally, the rest features are selected [11].

This research uses sklearn.feature_selection in Python to incorporate RFE. First, you need to select a machine-learning method to rank the importance of features. This research chose to use decision trees to score the importance of features and divided it into two steps. The first step was to find out how many features can be selected to obtain the highest accuracy rate. Taking this research database as an example, it can be shown that selecting 2 to 23 features will have different accuracy rates. We then determined the required features according to the accuracy rate. The second step was to show which features were selected. We used “support_” to show whether the features are true or false, which represent selected and unselected, respectively. We used “ranking_” to show the relative importance ranking of each feature; the selected feature is with a ranking of 1.

### 2.5. Construct A Prediction Model

#### 2.5.1. One-Dimension CNN

The core of convolutional neural network (CNN) is the convolutional layer, which is a neural network model that specializes in processing two-dimensional images, but it is also widely used in one-dimensional and three-dimensional data and has obtained favorable results. This study used Python’s Keras to construct the 1DCNN model and referred to the model architecture of Huang and Li [4]. There are 8 layers in total; the order is 1D convolutional layer, maximum pooling layer, 1D convolutional layer, maximum pooling layer, dropout layer, fully connected layer, dropout layer, and output layer, using Sigmoid activation function.

Model parameter settings: the number of convolution kernels (filter) was set to 16 and 32 in the two 1D convolutional layers; the convolution kernel size (kernel_size) was set to 3; the window size of the maximum pooling layer was set to 2; the stride was set to 1; padding was set to same, which means that the input data and output data remained the same size; and the dropout was set to 0.1. In addition, in this study, the parameter ranges of optimizer, epochs, and batch_size are shown in Table 3. Through interactive verification in Python combined with the grid search method (GridSearchCV) pairing, we obtained the best combination of 1DCNN model performance; and, finally, 5-fold cross-validation was used to evaluate the forecast results.

#### 2.5.2. Artificial Neural Network (ANN)

Artificial neural network is used in various fields and is suitable for classification and regression. It is similar to the structure of the human brain and consists of a large number of interconnected neurons. The basic structure is composed of an input layer, hidden layer, and output layer. This study used Keras in Python to construct an ANN prediction model. The network parameters of ANN are 24 variables in the input layer in the database; the number of neuron set in the hidden layer is 13; and the output layer is the outcome of the game (1/0). The parameter ranges of the initialization method (kernel_initializer), optimizer, epochs, and batch_size are shown in Table 4. GridSearchCV was used to optimize the performance of ANN model, which finally underwent 5-fold cross-validation to evaluate the prediction results.

#### 2.5.3. Support Vector Machine (SVM)

Support vector machine is one of the most popular machine-learning algorithms. It became very popular after it was developed in the 1990s [12]. SVM is suitable for binary classification, multi-classification, regression, etc. The concept is relatively simple and is mainly used to choose a hyperplane as the decision boundary, which can distinguish the variables according to their category (0 or 1).

This study used sklearn in Python for the SVM model. The parameter-range selection is shown in Table 5. In the kernel (kernel) part, linear and the popular nonlinear kernel-RBF (Gaussian radial basis function kernel) were used. The only parameter that affects the linear kernel is C; the parameters that affect RBF include C and gamma. GridSearchCV was used to optimize the performance of the SVM model, which finally underwent 5-fold cross-validation to evaluate the prediction results.

#### 2.5.4. Logistic Regression (LR)

Logistic regression began to be used in statistical software in the early 1980s and has gradually been widely used in academic research. It is one of the most popular binary classification machine-learning algorithms with simple algorithm and performs well in a wide range of applications. Logistic regression is similar to linear regression, both of which explore the relationship between the independent variable (*X*) and the response (*Y*). The difference is that the response (*Y*) in linear regression is a continuous variable, while the response discussed in logistic regression (*Y*) is the categorical variable (1 or 0), and no conditions are set for the probability distribution of the independent variable. If there are *n* independent variables, the logistic regression equation is as follows:

(3) $$\ln(\frac{P}{1-P})=\beta_{0}+\beta_{1}X_{1}+\beta_{2}X_{2}+\ldots +\beta_{n}X_{n}$$

where *P* is the probability of the event; (*P*/(1 − *P*)) is the odds ratio; *β*_0_ is the intercept or constant term; and *β*_1_, *β*_2_,..., *β_n_* are regression coefficients.

This study used Python to construct a logistic regression model. The parameter settings are shown in Table 6. There are 4 solvers (Solvers), namely liblinear, newton-cg, lbfgs, and sag. Among them, newton-cg, sag, and lbfgs support L2 regularization, while the liblinear solver supports L1 and L2 regularization. In addition, C is regularization strength, as in support vector machines, and smaller values specify stronger regularization. Finally, the same as the previous three models, GridSearchCV was used to search for the best parameter combination, and then we used 5-fold interactive verification to evaluate the prediction results.

### 2.6. Performance Indicators

This study used accuracy as one of the evaluation indicators for each prediction model, as it is the most commonly selected comprehensive indicator. A binary confusion matrix is generated in the win–loss prediction. The evaluation index can be obtained based on the actual and the predicted results. A true positive (*TP*) is correctly predicted as a win, and a false negative (*FN*) is incorrectly predicted as losing; false positive (*FP*) is incorrectly predicted as winning, and true negative (*TN*) is correctly predicted as losing. These four situations are shown in Table 7 to calculate the accuracy rate, as shown in Formula (4).

(4) $$Accuracy=\frac{TP+TN}{TP+FP+FN+TN}$$

Another evaluation indicator, AUC, represents the area under the receiver operator characteristic curve is selected. The ROC curve was developed in the 1950s for signal detection in radar echoes, and it has since been applied widely, especially to most unbalanced binary classification problems [13]. ROC space defines the false-positive rate (*FPR*) as the *x*-axis, and the true positive rate (*TPR*) as the *y*-axis. The formulas are as follows:

(5) $$FPR=\frac{FP}{FP+TN}$$

(6) $$TPR=\frac{TP}{TP+FN}$$

where *FPR* is the ratio of falsely judged as positive among all samples that are actually negative, and *TPR* is the ratio of all actually positive samples that are correctly judged to be positive. The area under the curve is AUC. The range of AUC is between 0 and 1, and the larger, the better.

### 2.7. Statistical Tests

Many studies use multiple models for analysis. How to compare the accuracy of each model and choose the best model are very important. In this study, ANOVA was performed to compare the accuracies among 1DCNN, ANN, SVM, and LR models before and after feature selection. T-test analysis was used to compare the performances before and after feature selection for ANN, SVM, and LR models.

## 3. Results

### 3.1. Before Feature Selection

Each team was organized into a dataset, with a total of 30 datasets. The data were first normalized and then divided into 80% training and 20% testing. Four prediction models with 5-fold cross-validation were built for each dataset. The prediction results for Texas Rangers (TEX) were selected as an example and described as follows.

#### 3.1.1. DCNN

**Figure 4:**
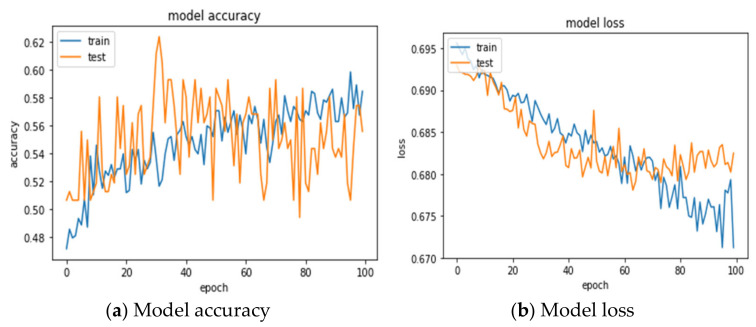
Training and testing process for 1DCNN (TEX).

The 24 variables of the original data were directly fed into the 1DCNN model, and the optimal parameter combination was searched by GridSearchCV. The 1DCNN with the combination of optimizer = rmsprop, epochs = 100, and batch_size = 20 obtains the best prediction accuracy (55.06 ± 2.04%), and AUC (0.5454 ± 0.01%). Table 8 shows the confusion matrix for the testing set presented by the 5-fold cross-validations. The confusion matrix with CV = 1 shows that, among the 162 matches, 41 were successfully predicted to win, 41 were misjudged, 47 were successfully predicted to lose, and 33 were misjudged. A total of 88 (41 + 47) matches were correctly predicted, and 74 (41 + 33) matches were wrongly predicted. An accuracy rate of 54.32% was obtained. The model accuracy and loss for training and testing process can be observed in Figure 4a,b.

#### 3.1.2. Artificial Neural Network (ANN)

**Figure 5:**
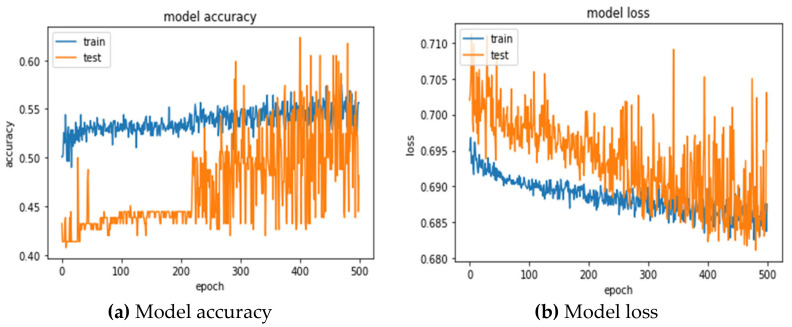
Training and testing process for ANN (TEX).

The hidden layer is set to 13 neurons in ANN model, and the optimal parameter combination is searched by GridSearchCV, which is a combination of kernel_initializer = lecun_uniform, optimizer = rmsprop, epochs = 500, and batch_size = 20. The best prediction accuracy and AUC are 52.22 ± 2.99% and 0.5480 ± 0.03, respectively. Table 9 is the confusion matrix presented by the 5-fold cross-validations, and model accuracy and loss for the training and testing process can be observed in Figure 5a,b.

#### 3.1.3. Support Vector Machine (SVM)

The optimal parameter combination was searched by using GridSearchCV, which is a combination of kernel = RBF, C = 1000, and gamma = 10. The best prediction accuracy and AUC are 64.79 ± 2.84% and 0.6500 ± 0.01, respectively. Table 10 is the confusion matrix presented by the 5-fold cross-validations.

#### 3.1.4. Logistic Regression (LR)

The optimal parameter combination was searched by using GridSearchCV, which is a combination of C = 10, penalty = L1, and solver = liblinear. The best prediction accuracy and AUC are 55.55 ± 2.21% and 0.5180 ± 0.01, respectively. Table 11 is the confusion matrix presented by the 5-fold cross-validations. The formula of LR is as follows:

(7) $$\begin{array}{cc} \ln\left(\frac{P}{1-P}\right)=0.764 & +0.000\times B_{1}+0.000\times B_{2}+0.000\times B_{3}+0.000\times B_{4}+\left(-1.088\right)\times B_{5}+0.270\times B_{6}\\ & +1.767\times B_{7}+\left(-8.760\right)\times B_{8}+3.868\times B_{9}+\left(-1.086\right)\times B_{10}+0.000\times B_{11}+0.000\times B_{12}\\ & +0.000\times B_{13}+0.000\times B_{14}+0.000\times B_{15}+2.812\times P_{1}+0.382\times P_{2}+0.000\times P_{3}\\ & +0.000\times P_{4}+1.322\times P_{5}+0.761\times P_{6}+0.000\times P_{7}+0.000\times P_{8}+\left(-1.787\right)X_{1} \end{array}$$

### 3.2. After Feature Selection

This study used RFE in Python to select features for each dataset. Different variables were selected for each dataset, and the selected ones were used for the ANN, SVM, and LR models. Take the Texas Rangers (TEX) team as an example; there were 12 selected variables, as listed in Table 12.

#### 3.2.1. Artificial Neural Network (ANN)

**Figure 6:**
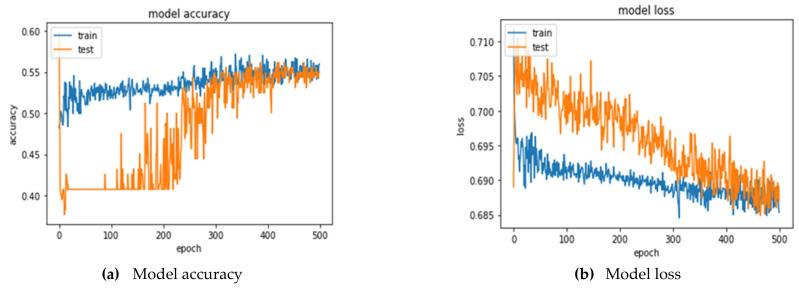
Training and testing process for ANN (TEX).

Twelve selected variables were used as the input, and the hidden layer was set to 7 neurons in the ANN model. The optimal parameter combination was searched by using GridSearchCV, which is a combination of kernel_initializer = he_normal, optimizer = adam, epochs = 500, and batch_size = 20. The best prediction accuracy and AUC are 53.70 ± 2.24% and 0.5709 ± 0.03, respectively. Table 13 is the confusion matrix presented by the 5-fold cross-validations. The model accuracy and loss for training and testing process can be observed in Figure 6a,b.

#### 3.2.2. Support Vector Machine (SVM)

The optimal parameter combination was searched by using GridSearchCV, which is a combination of kernel = RBF, C = 1000, and gamma = 10. The best prediction accuracy and AUC are 65.92 ± 2.80% and 0.6510 ± 0.02, respectively. Table 14 is the confusion matrix presented by the 5-fold cross-validations.

#### 3.2.3. Logistic Regression (LR)

The optimal parameter combination was searched by using GridSearchCV, which is a combination of C = 1000, penalty = L2, and solver = liblinear. The best prediction accuracy and AUC are 56.17 ± 1.56% and 0.5465 ± 0.02, respectively. Table 15 is the confusion matrix presented by the 5-fold cross-validations. The formula of LR after feature selection is as follows:

(8) $$\begin{array}{cc} \ln\left(\frac{P}{1-P}\right)=0.992 & +1.129\times B_{3}+1.576\times B_{4}+\left(-0.865\right)\times B_{6}+\left(-10.505\right)\times B_{8}+\left(-9.347\right)\times B_{12}\\ & +\left(-6.901\right)\times B_{14}+5.004\times B_{15}+2.450\times P_{1}+9.880\times P_{3}+7.034\times P_{5}+1.440\times P_{6}\\ & +\left(-2.433\right)X_{1} \end{array}$$

## 4. Discussion

The prediction accuracies before feature selection and after feature selection from 1DCNN, ANN, SVM, and LR models for each team are listed in Table 16 and Table 17, respectively. Table 18 compares the average prediction accuracies among four prediction models. The highest prediction accuracy (65.75%) was from SVM after feature selection. The prediction accuracies before and after feature selections from SVM models for 30 teams are greater than 60%, and the averages are around 65%. LR ranks as the second highest. The prediction accuracy of SVM is significantly different from the other three models before and after feature selections.

Comparisons with the state-of-the-art are shown in Table 19. As the relevant research on predicting the outcome of MLB matches uses data from different years and different lengths of time to make predictions, it is not possible to make fair comparisons. The same is that research objects are 30 MLB teams with data accumulation.

From the perspective of input variables, the selection of different variables will affect the prediction accuracy. Jia et al. [5] collected data related to scores and *Win%* for the three parts of 30 teams of beaters, pitchers, and teams, and finally got the best prediction accuracy rate of 59.60% after feature selection. Soto Valero [7] collected different game data from two websites, also including the win percentage for current season (won percentage for current season) and score-related variables. After feature selection, the best average prediction accuracy rate is 58.92%. Elfrink [6] collected data and expanded the five parts of the event time (day and night), home/away team, baseball field, enemy team, and day of the week, and then predicted the outcome of the game. The best average accuracy rate is 55.52%. The variables collected by Cui [8] include ELO, which can be used to explain the season performance over time. The best average prediction accuracy after feature selection is 61.77%. Data collection in our research focused on the performance of hitters, pitchers, and scoring, and, coupled with the variable *Win%*, we can obtain the best average prediction accuracy of 65.75% after feature selection. We found that only Elfrink [6] did not use score-related or win-rate-related variables; this choice may result in lower prediction accuracy. *Win%* is the only variable that is selected in the feature selection process for the 30 datasets in this study, meaning that *Win%* is vital in predicting the outcome of MLB next game.

From the perspective of the prediction method, the related literature uses a variety of machine-learning models with the variables after feature selection to predict the outcome of the game. It can be learned from Jia et al. [5], Soto Valero [7], and Cui [8] that the prediction results of 59.6% (SVM), 59% (SVM), and 61.77% (LR) are obtained, respectively. SVM and LR perform better in a variety of machine-learning models. In this study, four prediction models (1DCNN, ANN, SVM, and LR) are used to predict the outcome of the game for 30 datasets before and after feature selection. GridSearchCV is used to find the best combination of parameters to improve the performance of the model. The best prediction results are from SVM, followed by LR, and finally 1DCNN and ANN, echoing the prediction results of Jia et al. [5] and Soto Valero [7].

The difference between this study and other related works in the literature is that we collect game data from different baseball references, from which we can download game logs (Game Logs) according to different teams. The related literature uses the game logs downloaded by Retrosheet based on the home and away teams. This study expected to predict the outcome of the next game for 30 teams and performed the steps of feature selection to obtain the prediction accuracy. Therefore, it was more suitable and more convenient to collect data from Baseball-reference.com (assessed on 21 January 2021). This research conducted the feature selection for 30 teams individually to figure out the key variables that affect the outcome of each team.

## 5. Conclusions and Suggestions

This research collected the match data of 30 teams in the 2015–2019 seasons of MLB to predict and to improve the accuracy of predicting the outcome of the next MLB match. Four prediction models, namely 1DCNN, ANN, SVM, and LR, before and after feature selection for each team, were applied in this study. The average accuracies for thirty teams from 1DCNN, ANN, SVM, and LR models before feature selection were 55.48%, 54.29%, 64.25%, and 56.21%, respectively; the average accuracies for thirty teams from the 1DCNN, ANN, SVM, and LR models after feature selection were 55.48%, 54.47%, 65.75%, and 56.70%, respectively. SVM performs the best. This is consistent with the prediction results of related literatures. Notably, the individual highest accuracy, 70.74%, was found for team Baltimore Orioles from the SVM model after feature selection. The results show that the highest average accuracy (65.75%) and AUC (0.6501) were from SVM after feature selection. However, the difference between the accuracies before and after feature selection was found to be significant for the SVM model only, and not for the ANN and LR models.

The prediction was made for each team individually. The key variables and season performance of each team can provide some reference information for team managers, fans, and game enthusiasts, as well as for scholars in the field of sports prediction. They can be applied to different ball games, but it does not necessarily achieve the same predictive performance in the future.

Compared with the related literature, the contributions of this study are (1) that the prediction results before and after model feature selection are discussed; (2) the use of 1DCNN to construct a model to predict the outcome of the next game without feature selection; (3) that the prediction was made for each team individually; and (4) that, through the selection of variables and the setting of model parameters, the accuracy of prediction accuracy of the next MLB match was increased to more than 64%.

The limitations of this research are that (1) this research organizes 30 teams into individual datasets, and the amount of matches is relatively small; (2) only one feature-selection method (RFE) was used in the prediction model; (3) the dataset accumulation in this study was manually build, so it was more time-consuming and was easier to make mistakes; and (4) this research uses team data. The individual performance of the players or the season performance of the players can be considered to predict the outcome of the match in the future.

## Figures and Tables

**Table 1:** MLB variables.

Variable	Abbreviation	Variable	Abbreviation
*B1*	PA	*B13*	SLG
*B2*	AB	*B14*	OPS
*B3*	R	*B15*	LOB
*B4*	H	*P1*	H
*B5*	HR	*P2*	R
*B6*	RBI	*P3*	BB
*B7*	BB	*P4*	SO
*B8*	SO	*P5*	HR
*B9*	SB	*P6*	ERA
*B10*	CS	*P7*	AB
*B11*	BA	*P8*	WHIP
*B12*	OBP	*X1*	Win%

**Table 2:** Accumulation for MLB variables (HOU).

Year	Variable Game	*B1*	*B2*	*B3*	*…*	*P1*	*P2*	*P3*	*…*	*X1*	*Y*
**2015**	1	28	25	2	…	3	0	3	…	1.000	0
	2	61	55	2		10	2	3		0.500	0
	3	96	83	3		21	7	6		0.333	1
	…	…	…	…	…	…	…	…	…	…	…
	162	6073	5459	729	…	1308	618	423	…	0.531	1
**2016**	1	38	34	5	…	4	3	4	…	1.000	0
**…**	…	…	…	…	…	…	…	…	…	…	…
**2019**	161	6310	5540	906	…	1192	632	443	…	0.656	1
	162	6349	5573	912		1197	635	444		0.658	1

**Table 3:** Parameter setting for 1DCNN.

Parameter	Range
optimizer	Adam, RMSprop
epochs	50, 100, 300, 500
batch_size	10, 20, 30

**Table 4:** Parameter setting for ANN.

Parameter	Range
kernel_initializer	Zeros, RandomNormal, glorot_normal, glorot_uniform, he_normal, uniform, lecun_uniform, he_uniform
optimizer	Adam, RMSprop
epochs	50, 100, 300, 500
batch_size	10, 20, 30

**Table 5:** Parameter setting for SVM.

Parameter	Range
kernel	Linear, RBF
C	1, 10, 100, 1000
gamma	0.0001, 0.001, 0.1, 1, 10, 100, 1000

**Table 6:** Parameter setting for LR.

Parameter	Range
penalty	L1, L2
C	1, 10, 100, 1000
Solvers	Liblinear, newton-cg, lbfgs, sag

**Table 7:** Binary confusion matrix.

	Predicted		
Actual		Win	Lose
	Win	True Positive (*TP*)	False Negative (*FN*)
	Lose	False Positive (*FP*)	True Negative (*TN*)

**Table 8:** Confusion matrix for 1DCNN (TEX).

Predicted						
	CV		Win	Lose	Accuracy (%)	AUC
Actual	1	Win	41	41	54.32	0.5402
		Lose	33	47		
	2	Win	39	43	54.32	0.5402
		Lose	31	49		
	3	Win	41	41	52.47	0.5276
		Lose	36	44		
	4	Win	44	38	58.64	0.5686
		Lose	29	51		
	5	Win	48	34	55.56	0.5502
		Lose	38	42		
Average					55.06 (±2.04)	0.5454 (±0.01)

**Table 9:** Confusion matrix for ANN before feature selection (TEX).

Prediction Results						
	CV		Win	Lose	Accuracy (%)	AUC
Actual results	1	Win	38	58	50.00	0.5407
		Lose	23	43		
	2	Win	32	64	52.47	0.5502
		Lose	13	53		
	3	Win	52	44	56.79	0.5762
		Lose	26	53		
	4	Win	44	52	53.70	0.5691
		Lose	23	43		
	5	Win	21	75	48.15	0.5038
		Lose	9	57		
Average					52.22 (±2.99)	0.5480 (±0.03)

**Table 10:** Confusion matrix for SVM before feature selection (TEX).

Prediction Results						
	CV		Win	Lose	Accuracy (%)	AUC
Actual results	1	Win	39	36	61.11	0.6386
		Lose	27	60		
	2	Win	48	29	64.81	0.6493
		Lose	28	57		
	3	Win	49	26	63.58	0.6466
		Lose	33	54		
	4	Win	51	28	64.81	0.6493
		Lose	29	54		
	5	Win	52	31	69.75	0.6659
		Lose	18	61		
Average					64.79 (±2.84)	0.6500 (±0.01)

**Table 11:** Confusion matrix for LR before feature selection (TEX).

Prediction Results						
	CV		Win	Lose	Accuracy (%)	AUC
Actual results	1	Win	44	44	53.70%	0.5062
		Lose	31	43		
	2	Win	41	43	52.47%	0.5038
		Lose	34	44		
	3	Win	46	27	56.17%	0.5185
		Lose	44	45		
	4	Win	49	30	58.64%	0.5238
		Lose	37	46		
	5	Win	42	39	56.79%	0.5375
		Lose	31	50		
Average					55.55 (±2.21)	0.5180 (±0.01)

**Table 12:** Selected variables (TEX).

Team	Selected Variables
TEX	R(*B3*), H(*B4*), RBI(*B6*), SO(*B8*), OBP(*B12*), OPS(*B14*), LOB(*B15*), H(*P1*), BB(*P3*), HR(*P5*), ERA(*P6*), *Win%*(*X1*)

**Table 13:** Confusion matrix for ANN after feature selection (TEX).

Prediction Results						
	CV		Win	Lose	Accuracy (%)	AUC
Actual results	1	Win	45	51	55.56	0.5971
		Lose	21	45		
	2	Win	38	58	54.94	0.5919
		Lose	15	51		
	3	Win	34	62	49.38	0.5038
		Lose	20	46		
	4	Win	32	64	53.70	0.5696
		Lose	11	55		
	5	Win	37	59	54.94	0.5919
		Lose	14	52		
Average					53.70 (±2.24)	0.5709 (±0.03)

**Table 14:** Confusion matrix for SVM after feature selection (TEX).

Prediction Results						
	CV		Win	Lose	Accuracy (%)	AUC
Actual results	1	Win	47	30	60.49	0.6180
		Lose	34	51		
	2	Win	51	24	67.28	0.6313
		Lose	29	58		
	3	Win	50	25	67.90	0.6814
		Lose	27	60		
	4	Win	54	26	67.90	0.6814
		Lose	26	56		
	5	Win	58	24	66.05	0.6427
		Lose	31	49		
Average					65.92 (±2.80)	0.6510 (±0.03)

**Table 15:** Confusion matrix for LR after feature selection (TEX).

Prediction Results						
	CV		Win	Lose	Accuracy (%)	AUC
Actual results	1	Win	56	26	57.41	0.5618
		Lose	43	37		
	2	Win	43	38	54.32	0.5238
		Lose	36	45		
	3	Win	43	38	54.32	0.5238
		Lose	36	45		
	4	Win	42	37	56.79	0.5586
		Lose	33	50		
	5	Win	49	28	58.02	0.5645
		Lose	39	45		
Average					56.17 (±1.56)	0.5465 (±0.02)

**Table 16:** Prediction accuracies for four models before feature selection.

Number	Team	Accuracy (%)				*p*
		1DCNN	ANN	SVM	LR	
1	ATL	53.09 (±1.91)	52.72 (±1.59)	66.66 (±3.12) ^a^	55.18 (±2.58)	0.00 ^**^
2	CIN	60.74 (±0.92) ^b^	55.80 (±0.30) ^b,c^	61.98 (±3.23) ^c^	58.37 (±1.57)	0.00 ^**^
3	MIA	60.62 (±1.06)	60.62 (±4.54)	65.06 (±2.33) ^b^	59.26 (±2.37) ^b^	0.05 ^*^
4	NYM	54.44 (±1.76)	51.36 (±0.72)	63.46 (±2.26) ^a^	53.46 (±3.80)	0.00 ^**^
5	PHI	58.27 (±2.39)	57.53 (±1.53)	63.83 (±4.41)	59.88 (±2.84)	0.14
6	WSN	50.62 (±2.38)	49.38 (±0.00)	64.44 (±2.09) ^a^	56.05 (±3.48) ^a^	0.00 ^**^
7	MIL	52.71 (±1.15)	56.30 (±2.51)	62.84 (±2.00) ^a^	53.33 (±3.95)	0.00 ^**^
8	CHC	55.31 (±0.84)	52.47 (±0.00)	64.20 (±2.07) ^a^	59.63 (±2.16) ^a^	0.00 ^**^
9	STL	56.42 (±0.92)	55.56 (±0.00)	65.43 (±3.56) ^a^	54.57 (±3.11)	0.00 ^**^
10	PIT	57.65 (±1.39) ^b^	51.24 (±1.53) ^b^	66.54 (±1.53) ^a^	54.57 (±3.55)	0.00 ^**^
11	SFG	51.73 (±1.53)	50.49 (±0.46)	65.31 (±3.61) ^a^	53.21 (±2.35)	0.00 ^**^
12	SDP	55.80 (±0.84) ^b^	56.54 (±1.49) ^c^	66.55 (±3.99) ^b,c^	58.52 (±2.98)	0.01 ^*^
13	LAD	61.11 (±0.63)	60.49 (±1.03)	61.36 (±3.37)	59.50 (±2.09)	0.38
14	COL	53.33 (±2.01)	50.74 (±3.46) ^a^	62.59 (±3.09) ^a^	54.08 (±3.41)	0.01 ^*^
15	ARI	56.79 (±2.21) ^b^	51.36 (±0.91)	63.95 (±2.12) ^a^	51.07 (±2.65) ^b^	0.00 ^**^
16	TBR	54.20 (±2.80)	51.11 (±0.60)	63.83 (±2.64) ^a^	53.83 (±5.14)	0.00 ^**^
17	BOS	60.86 (±1.08) ^b^	54.44 (±1.26) ^b,c^	62.84 (±3.85) ^c,d^	57.11 (±1.47) ^d^	0.00 ^**^
18	NYY	59.50 (±1.08)	58.88 (±0.74)	65.43 (±1.41) ^a^	54.32 (±1.79) ^a^	0.00 ^**^
19	TOR	51.73 (±2.48)	49.14 (±2.43) ^b^	63.33 (±2.01) ^a^	56.29 (±2.48) ^b^	0.00 ^**^
20	BAL	58.03 (±2.50)	55.68 (±1.06) ^b^	62.22 (±3.77) ^b^	60.62 (±3.59)	0.03 ^*^
21	CHW	52.84 (±1.73) ^b^	57.28 (±0.91) ^b^	65.68 (±2.46) ^a^	54.57 (±3.26)	0.00 ^**^
22	CLE	51.61 (±1.00) ^b^	54.44 (±0.25)	62.96 (±2.40) ^a^	58.27 (±2.78) ^b^	0.00 ^**^
23	KCR	55.43 (±1.63)	55.43 (±1.37)	64.81 (±2.47) ^a^	56.30 (±2.80)	0.00 ^**^
24	DET	54.69 (±1.14) ^b,c^	60.25 (±1.08) ^b^	63.95 (±3.96) ^c^	61.48 (±3.55)	0.01 ^*^
25	MIN	56.17 (±1.10) ^b^	52.10 (±2.02) ^b^	63.21 (±3.23) ^a^	54.20 (±2.85)	0.00 ^**^
26	OAK	56.29 (±1.26) ^b^	55.19 (±2.55) ^c^	66.42 (±1.00) ^b,c^	59.78 (±3.46)	0.00 ^**^
27	HOU	57.90 (±0.72)	57.04 (±0.30)	63.09 (±3.61) ^a^	57.28 (±2.90)	0.01 ^*^
28	LAA	52.96 (±0.99)	52.47 (±1.0.3)	66.66 (±2.97) ^a^	52.47 (±1.51)	0.00 ^**^
29	SEA	48.40 (±1.49)	50.49 (±1.43)	64.20 (±2.53) ^a^	53.45 (±2.78)	0.00 ^**^
30	TEX	55.06 (±2.04)	52.22 (±2.44)	64.79 (±2.84) ^a^	55.55 (±2.21)	0.00 ^**^
Mean		55.48 (±3.22)	54.29 (±3.30) ^b^	64.25 (±1.47) ^a^	56.21 (±2.72) ^b^	0.00 ^**^

Note: ^*^ *p* < 0.05; ^**^ *p* < 0.01; model with superscript ^a^ is significantly different from the other three models; models with same superscripts ^b–d^ are significantly different from each other.

**Table 17:** Prediction accuracies for four models after feature selection.

Number	Team	Accuracy (%)				*p*
		1DCNN	ANN	SVM	LR	
1	ATL	53.09 (±1.91)	52.59 (±0.99)	66.05 (±2.47) ^a^	54.08 (±3.04)	0.00 ^**^
2	CIN	60.74 (±0.92)	55.56 (±0.00)	63.46 (±3.06) ^a^	60.49 (±2.18)	0.00 ^**^
3	MIA	60.62 (±1.06) ^b^	63.46 (±0.25) ^c^	65.53 (±0.99) ^b,d^	59.14 (±3.37) ^c,d^	0.00 ^**^
4	NYM	54.44 (±1.76)	51.48 (±0.84)	62.59 (±1.00) ^a^	52.47 (±3.38)	0.00 ^**^
5	PHI	58.27 (±2.39)	57.28 (±0.25) ^b^	64.20 (±2.84) ^b,c^	58.15 (±1.89) ^c^	0.01 ^*^
6	WSN	50.62 (±2.38)	49.38 (±0.00)	64.32 (±3.48) ^a^	55.56 (±1.17) ^a^	0.00 ^**^
7	MIL	52.71 (±1.15) ^b^	56.79 (±0.68) ^b^	65.31 (±3.80) ^a^	55.19 (±3.06)	0.00 ^**^
8	CHC	55.31 (±0.84)	52.47 (±0.00)	66.05 (±2.17) ^a^	60.74 (±2.80) ^a^	0.00 ^**^
9	STL	56.42 (±0.92)	54.82 (±0.46)	65.93 (±2.42) ^a^	56.30 (±2.42)	0.00 ^**^
10	PIT	57.65 (±1.39) ^b^	49.26 (±1.43) ^b^	66.91 (±2.39) ^a^	54.45 (±4.14)	0.00 ^**^
11	SFG	51.73 (±1.53)	50.62 (±1.41)	64.82 (±1.10) ^a^	51.86 (±2.51)	0.00 ^**^
12	SDP	55.80 (±0.84)	57.04 (±1.73)	67.53 (±1.38) ^a^	59.26 (±2.90)	0.00 ^**^
13	LAD	61.11 (±0.63) ^b^	60.99 (±0.25) ^c^	66.42 (±1.82) ^b,c^	60.24 (±3.61)	0.02 ^*^
14	COL	53.33 (±2.01)	50.62 (±2.10)	66.43 (±1.92) ^a^	54.44 (±1.81)	0.00 ^**^
15	ARI	56.79 (±2.21)	51.85 (±1.10)	63.95 (±3.57) ^a^	48.27 (±3.48)	0.00 ^**^
16	TBR	54.20 (±2.80)	50.62 (±1.87)	62.72 (±3.39) ^a^	52.10 (±3.26)	0.00 ^**^
17	BOS	60.86 (±1.08) ^b^	55.56 (±0.00) ^b^	65.80 (±3.67) ^a^	57.41 (±1.74)	0.00 ^**^
18	NYY	59.50 (±1.08)	59.51 (±0.74)	66.44 (±3.91) ^a^	60.62 (±3.30)	0.01 ^*^
19	TOR	51.73 (±2.48)	47.53 (±2.62) ^b^	66.54 (±1.81) ^a^	54.82 (±3.43) ^b^	0.00 ^**^
20	BAL	58.03 (±2.50)	56.54 (±0.74)	70.74 (±3.88) ^a^	61.48 (±3.07)	0.00 ^**^
21	CHW	52.84 (±1.73) ^b,c^	58.15 (±0.99) ^b^	62.59 (±2.64) ^c,d^	56.17 (±1.29) ^d^	0.00 ^**^
22	CLE	51.61 (±1.00)	54.07 (±0.50)	65.18 (±1.49) ^a^	59.51 (±3.26) ^a^	0.00 ^**^
23	KCR	55.43 (±1.63)	56.42 (±1.44)	66.05 (±1.83) ^a^	58.03 (±3.96)	0.00 ^**^
24	DET	54.69 (±1.14) ^a^	60.99 (±0.25) ^b^	64.81 (±1.95) ^b^	62.10 (±2.94)	0.00 ^**^
25	MIN	56.17 (±1.10) ^b^	51.73 (±1.58) ^b^	67.53 (±2.83) ^a^	54.94 (±5.24)	0.00 ^**^
26	OAK	56.29 (±1.26)	56.05 (±2.01)	66.43 (±4.02) ^a^	61.11 (±0.78) ^a^	0.00 ^**^
27	HOU	57.90 (±0.72)	57.28 (±0.46)	69.01 (±3.13) ^a^	60.74 (±2.42)	0.00 ^**^
28	LAA	52.96 (±0.99)	52.59 (±1.06)	67.53 (±4.07) ^a^	53.70 (±3.84)	0.00 ^**^
29	SEA	48.40 (±1.49)	48.89 (±2.32)	65.55 (±2.15) ^a^	51.36 (±3.03)	0.00 ^**^
30	TEX	55.06 (±2.04)	53.70 (±2.24) ^b^	65.92 (±2.80) ^a^	56.17 (±1.56)	0.00 ^**^
Mean		55.48 (±3.22)	54.47 (±3.91) ^b^	65.75 (±1.77) ^a^	56.70 (±3.52) ^b^	0.00 ^**^

Note: ^*^ *p* < 0.05; ^**^ *p* < 0.01; model with superscript ^a^ is significantly different from the other three models; models with same superscripts ^b–d^ are significantly different from each other.

**Table 18:** Accuracies before and after feature selection.

Feature Selection	Accuracy (%)				*p*
	1DCNN	ANN	SVM	LR	
No	55.48 (±3.22)	54.29 (±3.30) ^b^	64.25 (±1.47) ^a^	56.21 (±2.72) ^b^	0.00 **
Yes	55.48 (±3.22)	54.47 (±3.91) ^b^	65.75 (±1.77) ^a^	56.70 (±3.52) ^b^	0.00 **

Note: ** *p* < 0.01; model with superscript ^a^ is significantly different from the other three models; models with superscript ^b^ are significantly different from each other.

**Table 19:** Comparisons with related studies.

Author	Input Variables (After Feature Selection)	Methods	Accuracy (%)	AUC
Jia et al. [5]	BA, RBI, OBP, ERA, H, E, and *Win%* for each team	**SVM** AdaBoost LogitBoost	59.60	-
Soto Valero [7]	isHomeClub, Log5, PE, WP, RC, HomeWonPrev, VisitorWonPrev, BABIP, FP, PitchERA, OBP, SLG, VisitorLeague, HomeVersusVisitor, Stolen	**SVM** ANN Decision Tree k-NN	58.92	-
Elfrink [6]	AB, AVG, OBP, SLG, OPS, BA/RISP, WHIP, RA	Random forest Linear model **XGBoost**	55.52	-
Cui [8]	OBP, ISO, FIP, WHIP, K/9, HR/9, K/BB, ELO, rest days between games	**LR** SVM k-NN Decision tree Random forest XGBoost	61.77	0.6706
This study	TEX Team R, H, RBI, SO, OBP, OPS, LOB, H, BB, H, ER, *Win%*	1DCNN ANN **SVM** LR	65.75	0.6501

Methods with bold format achieved the highest accuracy in the study.

## Data Availability

MLB game data were collected from Baseball-reference website (https://www.baseball-reference.com (assessed on 21 January 2021)).
